# Active Antioxidant Packaging from Essential Oils Incorporated Polylactic Acid/Poly(butylene adipate-co-terephthalate)/Thermoplastic Starch for Preserving Straw Mushroom

**DOI:** 10.3390/foods11152252

**Published:** 2022-07-28

**Authors:** Hang Gui, Meiyan Zhao, Shiqi Zhang, Ruoyu Yin, Changying Hu, Min Fan, Li Li

**Affiliations:** 1 College of Food Science and Technology, Shanghai Ocean University, Shanghai 201306, China; gh1782804268@163.com (H.G.); zmy2911009857@163.com (M.Z.); zhangsqheher666@163.com (S.Z.); 15216818263@163.com (R.Y.); mfan@shou.edu.cn (M.F.); 2 Department of Food Science and Engineering, Jinan University, Guangzhou 510632, China

**Keywords:** active packaging, peppermint oil, clove oil, antioxidant activity, straw mushroom

## Abstract

The short-term shelf life of straw mushrooms (*Volvariella volvacea*) is a major challenge, hampering their wide distribution. Thus, the aim of this work was to develop a novel active packaging composed of essential oils (EOs), particularly clove oil (CO) and peppermint oil (PO), to reduce autolysis of straw mushrooms. The morphological characterizations, mechanical properties, barrier properties, and antioxidant activities of the films were characterized. The suppressive effects of the EOs on straw mushroom autolysis were estimated during storage at 16 ± 1 °C for 96 h. The results indicated that the addition of EOs weakened the mechanical and barrier properties of the films. The radical-scavenging activities of polylactic acid/poly(butylene adipate-co-terephthalate)/thermoplastic starch-peppermint oil (PLA/PBAT/TPS-PO) and polylactic acid/poly(butylene adipate-co-terephthalate)/thermoplastic starch-clove oil (PLA/PBAT/TPS-CO) films for 2,2-diphenyl-1-picrylhydrazyl were 56.0% and 91.3%, respectively. However, the PLA/PBAT/TPS-PO film was more effective in reducing polyphenol oxidase activity and maintaining the total phenol content of straw mushrooms, demonstrating better antioxidative activity. Mushrooms packaged with the PLA/PBAT/TPS-PO film exhibited the lowest autolysis rate (42.29%, *p* < 0.05) after 96 h of storage. Thus, PO is a good preservative agent for straw mushroom.

## 1. Introduction

Straw mushroom (*Volvariella volvacea*) is widely grown in China [1]. Straw mushroom is nutritious and rich in protein (3.9% protein on a fresh weight basis). Autolysis of the fruit body at below 10 °C and high levels of water content (~90% w.b.) at above 25 °C limit the shelf life of straw mushrooms to 1–2 d after harvest [2,3,4]. An effective technique for preserving straw mushrooms is urgently needed.

The conventional methods, such as cold storage and modified atmosphere packaging (MAP), are not suitable to preserve straw mushroom because it is sensitive to low temperatures [1]. Li et al. proposed a combined method by using 10-min ultrasound treatment (300 w power) and 95% relative humidity (RH) [4], which extended the postharvest quality of straw mushrooms from 24 h or 48 h to 72 h. Dhalsamant et al. [2] reported that perforation mediated MAP effectively prolonged the shelf life of straw mushrooms treated with CaCl_2_ (0.5%) to 6 days at 12 ± 1 °C.Meanwhile, these methods have high requirements for equipment.

Active packaging is effective in maintaining food quality and enhancing food safety [5]. This work on active packaging mainly focuses on adding antioxidant and antibacterial agents directly into packaging materials. This type of active packaging belongs to the transferable active packaging. Natural antioxidant and antibacterial agents from essential oils (EOs) have eminent biological properties and are thus widely used [6]. Natural products from aromatic plants, including EOs, are attracting considerable interests [7]. Peppermint oil (PO) and clove oil (CO), as natural antioxidant and antibacterial substances, are good alternatives to synthetic antioxidants and antibacterial agents for food packaging. PO obtained from peppermint plants is an important EO. The major chemical components of PO are menthol (33–60%) and menthone (15–32%). These chemical components have antibacterial and antioxidant properties [8,9,10]. Menthol and some monoterpenes can penetrate into cell membranes and, as a result, the function of lipid biolayers is intensively negatively altered [11]. It has been reported that PO can maintain membrane integrity and delay senescence in fruit [8]. Qu et al. [8] concluded that PO fumigation is a promising method to inhibit polyphenol oxidase (PPO) activity and improve the quality of button mushrooms. CO, isolated from clove plants, contains mainly eugenol (76.8%) [12]. The phenolic hydroxyl group of eugenol is the main functional group with antibacterial and antioxidant effects. Eugenol achieves its antioxidant effect by contributing hydrogen atoms to trap oxygen atoms and reduce free radical production [13]. Previously, it has been reported that CO can enhance antioxidant capacities in various kinds of fruit and vegetables, such as shiitake mushrooms [14] and mangoes [15].

In our previous experiment [16], we found that the polylactic acid/poly (butylene adipate-co-terephthalate)/thermoplastic starch (PLA/PBAT/TPS) film with 14 wt% PLA, 56 wt% PBAT, and 30 wt% TPS had a stronger inhibitory effect against autolysis in straw mushrooms than films containing low or high amounts of TPS. The antioxidant activities of active packaging films have been extensively studied [17,18]. However, to the best of our knowledge, the effect of active antioxidant packaging composed of EOs on the response to straw mushroom autolysis has not been studied and compared in great detail [1,2,3,4]. The objective of this study was to evaluate the effects of EOs (including PO and CO) on the quality preservation of straw mushroom during storage at 16 ± 1 °C. The antioxidant capacities of the tested films containing PO and CO and the antioxidant effects produced during the preservation of straw mushrooms were measured. The results were expected to provide insights into the application of EOs to food storage. 

## 2. Materials and Methods

### 2.1. Materials

Straw mushrooms, cornstarch (Reagent grade), PLA 721, PBAT A400, and chain extender (ADR4370F, molecule weight: 6800, epoxy equivalent: 285 g mol^−1^) were supplied in accordance with Zhao et al. [16]. CO (AR, ≥80%, GC) was purchased from Macleans Biochemical Technology Co., Ltd. (Shanghai, China). PO (proportion: 100%) was purchased from Luyuan Natural Spice Oil Refinery (Ji’an, China). 

### 2.2. Film Preparation

Films were prepared by the melt blending method described by Zhao et al. [16] with some modifications. 30 wt% TPS, 14 wt% PLA, and 56 wt% PBAT, EOs (PO or CO, 5 wt%) [19], and 1 wt% chain extender were mixed. Three kinds of films were marked: PLA/PBAT/TPS, PLA/PBAT/TPS-PO, and PLA/PBAT/TPS-CO. Each experimental film had an average thickness of 40 ± 3 μm.

### 2.3. Characterization of Film

#### 2.3.1. Mechanical Properties

Tensile strength (TS) and elongation at break (EB) were determined using an electronic tensile testing machine (XLW-EC, Labthink, Jinan, China) in accordance with D882-12 [20].

#### 2.3.2. Morphological Characterization

The microstructures of the fracture surface and surface of the films were observed with a scanning electron microscope (SEM, Hitachi SU5000, Hitachi, Japan) in accordance with the method described by Zhao et al. [16].

#### 2.3.3. Water Vapor Transmission Rate (WVTR) and Oxygen Transmission Rate (OTR)

WVTR was determined by a water vapor transmission rate tester (W-B-31E, Labstone, Wuzhou, Guangxi, China) in accordance with the standard of ASTM E398-13 [21]. 

OTR of the films was measured using a gas permeation meter (PERME G2/132, Labthink Instruments Co., Ltd., Jinan, China) in accordance with the standard of D1434-82 [22].

#### 2.3.4. Antioxidant Capacity

The antioxidant capacity of each film was measured using DPPH in accordance with the method described by Eliezer et al. [23]. Antioxidant activity was expressed as percentage.

### 2.4. Treatment of Straw Mushrooms

After harvest, straw mushrooms were transferred at 16 ± 1 °C to the laboratory within 2 h. Straw mushrooms with no mechanical damage were selected, and the samples were packed in rectangular bags (27 × 30 cm) constructed from the tested films and stored at 16 ± 1 °C for 96 h. The mushrooms, varying from 470 g to 500 g in weight, were randomly divided into four treatment groups: (1) control group (no film packaging); (2) PLA/PBAT/TPS group; (3) PLA/PBAT/TPS-PO group; (4) PLA/PBAT/TPS-CO group. 

### 2.5. Qualities of Straw Mushrooms

#### 2.5.1. PPO and Total Phenol Content (TPC)

PPO was determined according to the methods of Zhao et al. [16]. The crystal structure of PPO (Protein Data Bank ID: 2P3X) was obtained online (http://www.rcsb.org/pdb, accessed on 13 October 2020) according to the method of Chen et al. [12]. The enzyme structure was prepared by using UCSF Chimera, production version 1.14 software (Copyright (c) 2000–2019 by the Regents of the University of California, Berkeley, CA, America). TPC was determined at a wavelength of 280 nm according to the method of Cao et al. [24]. 

#### 2.5.2. Moisture Status

The moisture status of the straw mushrooms was analyzed through low-field nuclear magnetic resonance (LF-NMR, NMI20, Shanghai) according to the method of Zhao et al. [16]. Spin–spin relaxation time (T2) image was obtained with the NMR relaxation time inversion and fitting software. The images were obtained through magnetic resonance imaging (MRI).

#### 2.5.3. Autolysis Rate

The autolysis rate of straw mushrooms was calculated according to the percentage of autolyzed straw mushrooms in each group.

#### 2.5.4. Rate of Weight Loss (RWL)

The weight of straw mushrooms was measured with an electronic balance. The weight of all straw mushrooms from each group was measured once a day. The RWL (%) was calculated by Formula (1):

(1) $$\mathrm{RWL}\;(\%)=\frac{W_{0}-W_{1}}{W_{0}}$$

where *W*_0_ is the initial weight (g) of straw mushrooms, and *W*_1_ is the final weight (g) of straw mushrooms.

#### 2.5.5. Hardness

Fruit hardness is defined as the pressure (N) that a unit area of fruit can withstand [24]. The hardness of the straw mushrooms was measured by a GY-4 fruit hardness tester (GY-4, Zhejiang Top Yunong Technology Co., Ltd., Hangzhou, China). Three straw mushrooms were randomly selected from each group and three different parts of each straw mushroom weremeasured. The average hardness was calculated.

#### 2.5.6. Total Soluble Solids (TSS)

Straw mushrooms (10 g) were ground and filtered with three layers of gauze. TSS content in the juices was measured with an Abbe refractometer (LH-T32, Lohand Biotech, Hangzhou, China) and expressed on the Brix scale. 

#### 2.5.7. Microbiological Analysis

Microbiological analysis was carried out according to the method of Kshanaprava et al. [3] with some modifications. Straw mushrooms (5 g) were serially diluted (1:10), and a 0.1 mL aliquot of each sample was placed in a medium for culture. The colonies of the microorganisms in the nutrient agar medium were counted after 2 d at 37 °C. 

### 2.6. Statistical Analyses

All statistical analyses were performed with SPSS (IBM 2019, SPSS Statistics 26, International Business Machines Corp., Armonk, NY, USA) and Origin (OriginPro 2017C, OriginLab Corporation, Armonk, NY, USA). All data were calculated using Duncan’s multiple comparison tests and one-way analysis of variance.

## 3. Results and Discussion

### 3.1. Film Characterization

#### 3.1.1. Mechanical Properties

**Figure 1:**
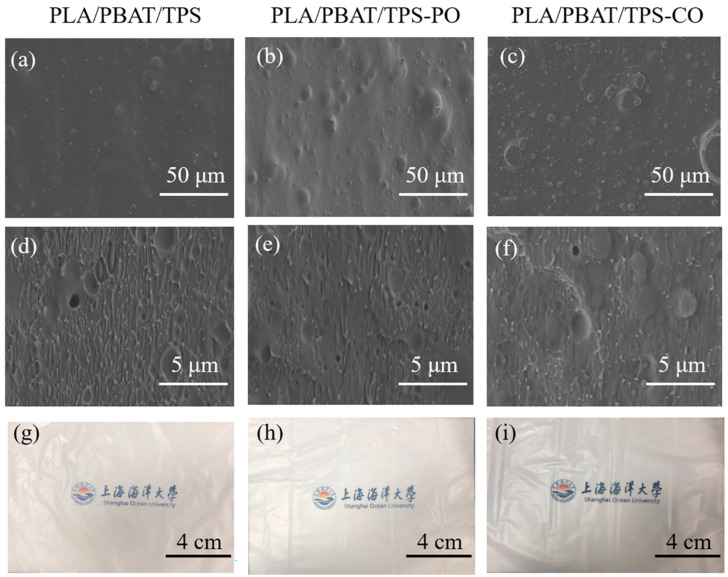
Scanning electron microscopy (SEM) images and photographs of the active films ((**a**–**c**) surface and (**d**–**f**) fracture surface of SEM micrographs; (**g**–**i**) photographs of the films). (**a**,**d**,**g**) PLA/PBAT/TPS film; (**b**,**e**,**h**) PLA/PBAT/TPS-PO film; (**c**,**f**,**i**) PLA/PBAT/TPS-CO film.

The EOs weakened the mechanical properties of the films (Table 1). The PLA/PBAT/TPS-CO film had the weakest TS (5.40 MPa), and a significant difference (*p* < 0.05) in TS was observed between the PLA/PBAT/TPS-PO and PLA/PBAT/TPS-CO films. In addition, the PLA/PBAT/TPS-PO film had the lowest EB (150%). The addition of EOs can interact with starch and cause the hydrogen bond between starch chains to be replaced by EOs. EOs can cause phase separation in a film and reduce its mechanical properties [25]. Azevedo et al. [26] had similar observations. They observed that the rigidity and strength of TPS/whey protein isolate nanocomposites decreased after the addition of rosemary EOs. This result was consistent with the film microstructure shown in Figure 1.

#### 3.1.2. Morphological Characterization

The scanning electron images (surface and fracture surface) of the different active films are shown in Figure 1. The films exhibited an extremely smooth surface. This indicated that PLA, PBAT, TPS and EOs had good compatibility (Figure 1g–i). As shown in the SEM surface images (Figure 1a–c), the EOs can cause phase separation in a film and increased the roughness of the film surface. The SEM images (Figure 1d–f) of the fracture surface showed that the EOs weakened the compactness of the films. In addition, the films with EOs presented rougher structures than the control films. Brandelero et al. [27] reported that films with large amounts of EOs had cracks and micropores. The diameters of the micropores formed in the control films without EOs were smaller than those formed in the films containing EOs.

#### 3.1.3. WVTR and OTR

As revealed in Table 2, the WVTR of the PLA/PBAT/TPS-PO and PLA/PBAT/TPS-CO films was significantly higher (*p* < 0.05) than the WVTR of the PLA/PBAT/TPS film. However, no significant difference in WVTR (*p* > 0.05) was found between the PLA/PBAT/TPS-PO and PLA/PBAT/TPS-CO films, which had similar trends with regard to changes in OTR. The microstructures of the films can affect their water-blocking efficiency [28]. As indicated in the SEM images of the films in Figure 1, the addition of EOs reduced the structural compactness of the blended films, facilitating water vapor transfer through the film [5]. Marzieh et al. [29] prepared a starch film containing Pistacia atlantica gum EOs and showed that the WVTR of the film containing EOs increased relative to that of the control film.

As listed in Table 2, the films with active ingredients had higher OTRs than the films without EOs. However, no significant difference (*p* > 0.05) in OTR was found between the PLA/PBAT/TPS-PO and PLA/PBAT/TPS-CO films. As shown in Figure 1, the film structure became increasingly heterogeneous after the addition of EOs, which facilitated oxygen transmission through the films and decreased oxygen barrier properties [30]. According to Aguilar-Sanchez et al. [31], the OTR of starch edible films with nanocomposites and Mexican oregano EOs significantly increased (*p* < 0.05) with respect to the control film.

#### 3.1.4. Antioxidant Capacity

As shown in Table 3, DPPH scavenging activity significantly varied (*p* < 0.05) among the film samples, and the highest activity was observed in the PLA/PBAT/TPS-CO film. Biddeci et al. [32] reported that the inhibition percentage of the free radical DPPH by PO released from the film was 41%. Gulcin et al. [33] concluded that the scavenging effect of CO on the DPPH radical was 83.6% at a concentration of 45 µg mL^−1^. In general, EOs have phenolic compounds that act as antioxidants that are highly reactive to peroxyl radicals, and the total phenolic content of CO is significantly higher (*p* < 0.05) than that of PO [34]. These findings can explain the high DPPH scavenging activity of CO.

### 3.2. Quality of Straw Mushrooms

#### 3.2.1. PPO and TPC

**Figure 2:**
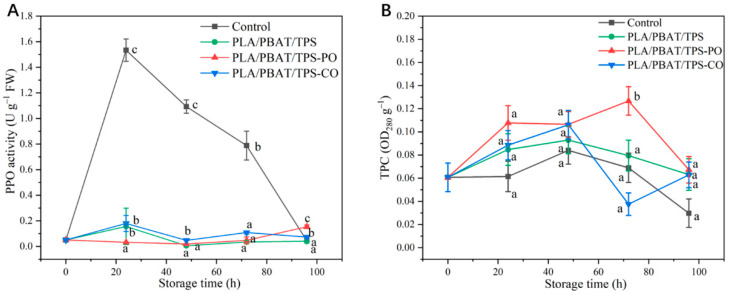
The effect of different packages on PPO activity (**A**) and TPC (**B**) of straw mushrooms. Different letters indicate significant differences within each parameter (*p* < 0.05).

PPO plays a critical role in catalyzing the hydroxylation of phenols [8]. The mushrooms in the control group had the highest PPO activity (*p* < 0.05) before 96 h of storage (Figure 2A). PPO activity was inhibited in all the packaged mushrooms. Especially, the PPO activity of mushrooms packaged with the PLA/PBAT/TPS-PO film did not have a remarkable upward trend before 72 h of storage. The highest PPO activity of all other groups was observed at the 24th hour. PPO has an active site located at the core of its helix bundles, in which two copper ions complexed by the histidine residues are presented, and one histidine is engaged in a thioether linkage with a cysteine residue [12,35]. The main components (such as menthol or eugenol) of EOs (PO and CO) were embedded in the spiral component of the PPO structure and interacted with the key substrate-binding residues of PPO active sites [8,12]. Our results confirmed that PO can inactivate the PPO activity of mushrooms more effectively than CO.

As shown in Figure 2B, the TPC of mushrooms in the control and packaged groups first increased and then gradually decreased. A similar trend was observed by Liu et al. [36]. The mushrooms in the PLA/PBAT/TPS-PO group had the highest TPCs before 96 h of storage (Figure 2B). This result can be attributed to phenol oxidation by PPO. The PPO activities of mushrooms in the PLA/PBAT/TPS-PO group was low (Figure 2A). The accumulated phenols showed antioxidant activity, which may have contributed to the alleviation of browning in the mushrooms and maintained membrane integrity [36]. In addition, given that phenol compounds can be decomposed by the hydrogen of water, increase in the water content of mushrooms may decrease TPC [37].

PO has a more positive effect that inhibits PPO activity and maintains TPC than CO. Thus, the antioxidant capacity of PO was more suitable for straw mushrooms.

#### 3.2.2. Moisture Status (LF-NMR and MRI)

**Figure 3:**
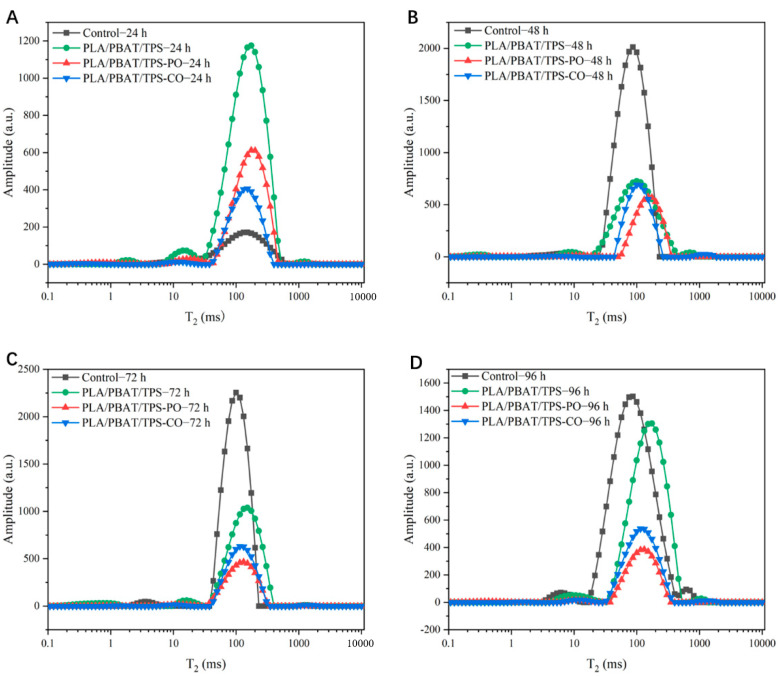
T_2_ relaxation time spectrum of straw mushrooms in different packages during storage. (**A**–**D**) relaxation time and signal amplitude at 24, 48, 72, and 96 h, respectively.

The T_2_ inversion maps of the straw mushrooms are shown in Figure 3. The peak area was expressed as the relative content of water [38]. The smallest T_23_ (100–1000 ms) peak area of mushrooms packaged with the PLA/PBAT/TPS-PO film was obtained after 48 h of storage, indicating that these mushrooms had the lowest free water content. When straw mushrooms were stored, their maturity accelerated, and free water increased rapidly. This stage encompassed the occurrence of autolysis. Then, the straw mushrooms were seriously corrupted, and the cells were destroyed as the storage period increased [39]. PO has antioxidant activity, maintains cell integrity, and delays senescence in the mushroom [7]. According to the results presented in Figure 3, PO was more effective in decreasing the free water content of the straw mushroom during postharvest storage.

**Figure 4:**
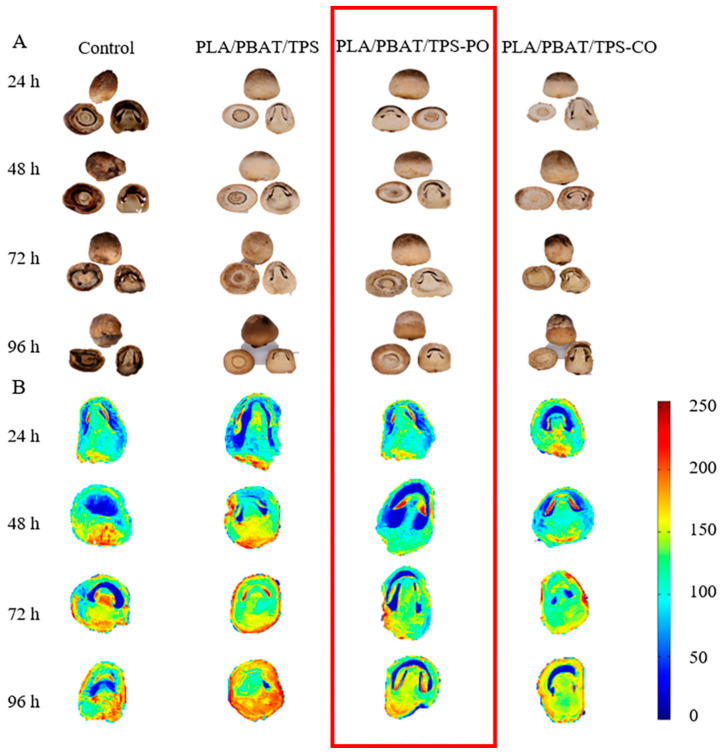
The effect of different packages on the physical appearance (**A**) and MRI (**B**) of straw mushrooms.

The pseudo-color maps of the MRI analysis results of the straw mushrooms are shown in Figure 4B. As shown in Figure 4B, the pseudo-color map of the mushrooms gradually changed from blue to red as storage time increased, indicating that the water content of the mushrooms gradually increased [40]. The colors of the PLA/PBAT/TPS-PO and PLA/PBAT/TPS-CO groups started to turn red at a later time relative to the PLA/PBAT/TPS group. The pseudo-color pictures of the mushrooms packaged with the PLA/PBAT/TPS-PO film were lighter than those of the PLA/PBAT/TPS-CO group. These results were consistent with Figure 4A.

#### 3.2.3. Autolysis Rate and RWL

**Figure 5:**
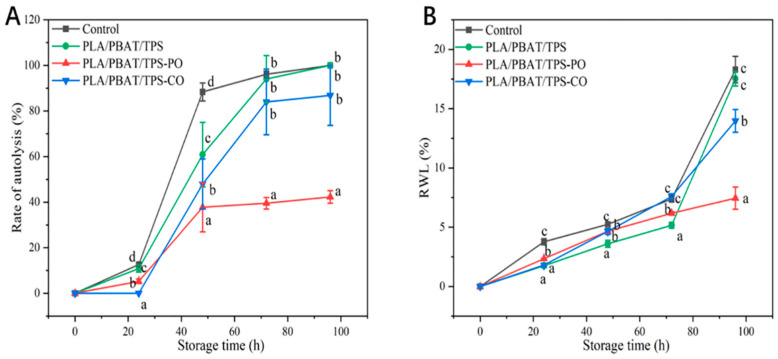
Effects of different packages on the rate of autolysis (**A**) and RWL (**B**) of straw mushrooms. Different letters indicate significant differences within each parameter (*p* < 0.05).

The mushrooms packaged with the PLA/PBAT/TPS-PO film exhibited the lowest rates of autolysis (*p* < 0.05) after 48 h of storage, and the autolysis rates of the mushrooms in the PLA/PBAT/TPS-PO group increased to 42.3% at the end of the storage period (Figure 5A). The autolysis rates of the mushrooms packaged with the film were mainly related to environmental humidity [41]. The PLA/PBAT/TPS film had the lowest WVTR (916 g m^−2^ 24 h^−1^, *p* < 0.05), and too much moisture in the packaging bag was not removed in time. Thus, the humidity of the microenvironment in the PLA/PBAT/TPS package was higher. Furthermore, EOs can inhibit enzyme activity, especially PO. Figure 2A indicates that PO was the most effective in inhibiting PPO activity. This finding can explain the lower autolysis rates of the mushrooms in the PLA/PBAT/TPS-PO group in the entire storage period. These results are consistent with the free water content of straw mushrooms in Figure 3 and Figure 4.

After 72 h of storage, the RWL of the mushrooms packaged with the PLA/PBAT/TPS-PO film showed the strongest inhibitory effect (*p* < 0.05) (Figure 5B). Only the RWL of mushrooms packaged with the PLA/PBAT/TPS-PO film was within the acceptable range of the market values (5–10%) after 96 h of storage [36]. The packaged mushrooms had a lower RWL than the control group, possibly because the films acted as effective barriers between surrounding environments and surfaces and eventually reduced water loss in the mushrooms [42]. Furthermore, mushrooms gradually matured after harvest and showed increased enzyme activity, which increased cell membrane permeability, promoted the extravasation of cell fluid and water [41], and increased the RWL of the mushrooms. Thus, the PO-induced decrease in autolysis rate (Figure 5A) might explain the inhibition of the RWL of the straw mushrooms in the PLA/PBAT/TPS-PO group.

#### 3.2.4. Hardness and TSS

**Figure 6:**
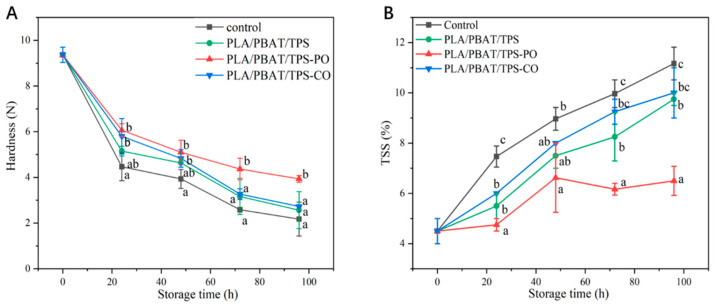
Effect of different packages on hardness (**A**) and TSS (**B**) of straw mushrooms. Different letters indicate significant differences within each parameter (*p* < 0.05).

Hardness is related to the maturity of fruit and vegetables [43]. In the present study, the hardness of the test samples declined during storage (Figure 6A). The hardness of the mushrooms packaged with the PLA/PBAT/TPS-PO film was higher than those in the other groups, especially after 48 h of storage. At 96 h of storage, the highest level (3.93 N) was obtained with the PLA/PBAT/TPS-PO film. Alteration in hardness is often related to starch oxidation, which increases the sugar content and water loss and reduces turgor pressure [7]. PO had a better antioxidant activity than CO for straw mushrooms (Figure 2A). These results confirm that the PLA/PBAT/TPS-PO film was the most effective in preserving the texture of the straw mushrooms.

TSS in fruit and vegetables can directly reflect maturity and quality [24]. The TSS of straw mushrooms is shown in Figure 6B. TSS increased during storage. The addition of EOs to packaging could inhibit the activity of the enzyme, which could be applied to slow down the increment of sugar content during storage. It might also be controlled by retarding its respiration, evapotranspiration. In contrast, the increase in sugar contents in the untreated fruit could be due to the accumulation of sugar due to hydrolysis of insoluble polysaccharides (starch) into simple sugars and the degradation of the cell walls, leading todissolved. Similar results were presented by Owolabi et al. [7], who reported an increase in the TSS values of mangosteen after storage. The TSS value of the PLA/PBAT/TPS-PO group was lower than the values of the other groups during storage. Meanwhile, the hardness of the mushrooms in the PLA/PBAT/TPS-PO group was the highest (Figure 6A). These results showed that the mushrooms in the PLA/PBAT/TPS-PO group had the lowest senescence.

#### 3.2.5. Microbiological Analysis

**Figure 7:**
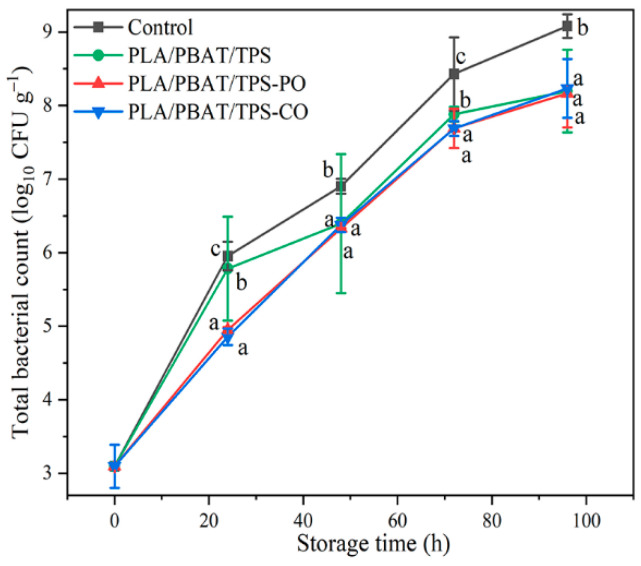
Total bacterial count of the straw mushrooms. Different letters indicate significant differences within each parameter (*p* < 0.05).

The film was effective in decreasing the total bacterial counts of mushrooms because it prevented contact between the mushrooms and dust or bacteria in the air. The addition of PO and CO to the PLA/PBAT/TPS films resulted in the significant decrease (*p* < 0.05) in the bacterial counts of mushrooms relative to the bacterial counts in the control and PLA/PBAT/TPS groups before 48 h of storage (Figure 7). However, no significant difference (*p* > 0.05) in total bacterial count was found between the PLA/PBAT/TPS-PO and PLA/PBAT/TPS-CO groups during storage. The EOs may have increased the antimicrobial properties as they were composed of bioactive aromatic compounds with antioxidant activities that control the level of oxidative damage [44].

## 4. Conclusions

In this work, active antioxidant packaging composed of EOs (PO and CO) on the response to straw mushroom preservation was studied and compared. Results demonstrated that EOs had a remarkable effect on the quality preservation of mushrooms. While the addition of EOs weakened the mechanical and barrier properties of the films, there was on the other hand a remarkable increase in the antioxidant activities of the films. The PLA/PBAT/TPS-CO film had a higher DPPH scavenging activity (91.3%) than that of the PLA/PBAT/TPS-PO film (56.0%). However, the PLA/PBAT/TPS-PO film had a better antioxidant activity for straw mushrooms that inhibited enzymatic activity in the mushrooms and thus showed reduced PPO activity and maintenance of TPC during the storage period. The PLA/PBAT/TPS-PO film was the most effective in inhibiting the autolysis of straw mushrooms and extended postharvest shelf lives to approximately 96 h during storage at 16 ± 1 °C. Further studies on the preservation mechanism of EOs for food during storage are needed to expand the application range of EOs.

## Figures and Tables

**Table 1:** Mechanical properties of films.

Sample	TS (MPa)	EB (%)
PLA/PBAT/TPS	8.54 ± 0.29 ^a^	235 ± 9.80 ^b^
PLA/PBAT/TPS-PO	6.53 ± 0.47 ^b^	150 ± 12.0 ^a^
PLA/PBAT/TPS-CO	5.40 ± 0.83 ^c^	167 ± 17.9 ^a^

Error bars indicate standard error (±SE). ^a–c^ Different superscripts indicate significant differences within each parameter (*p* < 0.05).

**Table 2:** WVTR and OTR of films.

Sample	WVTR (g m^−2^ 24 h^−1^)	OTR (cm^3^ m^−2^ day^−1^ 0.1 MPa^−1^)
PLA/PBAT/TPS	916 ± 58.1 ^a^	976 ± 2.73 ^a^
PLA/PBAT/TPS-PO	1036 ± 59.9 ^b^	1037 ± 64.9 ^ab^
PLA/PBAT/TPS-CO	1082 ± 61.8 ^b^	1130 ± 63.7 ^b^

Error bars indicate standard error (±SE). ^a,b^ Different superscripts indicate significant differences within each parameter (*p* < 0.05).

**Table 3:** DPPH of films.

Sample	DPPH (%)
PLA/PBAT/TPS	14.9 ± 0.8 ^a^
PLA/PBAT/TPS-PO	56.0 ± 7.8 ^b^
PLA/PBAT/TPS-CO	91.3 ± 1.5 ^c^

Error bars indicate standard error (±SE). ^a–c^ Different superscripts indicate significant differences within each parameter (*p* < 0.05).

## Data Availability

Data is contained within the article.
